# Association of Methylenetetrahydrofolate Reductase C677T Gene Polymorphisms with Mild Cognitive Impairment Susceptibility: A Systematic Review and Meta-Analysis

**DOI:** 10.1155/2021/2962792

**Published:** 2021-09-18

**Authors:** Jiahui Sun, Xuefan Jiang, Ming Zhao, Lina Ma, Hui Pei, Nanyang Liu, Hao Li

**Affiliations:** ^1^Graduate School, Beijing University of Chinese Medicine, Beijing, China; ^2^Department of Geratology, Xiyuan Hospital, China Academy of Chinese Medical Sciences, Beijing, China; ^3^Wangjing Hospital, China Academy of Chinese Medical Sciences, Beijing, China

## Abstract

**Background:**

Methylenetetrahydrofolate reductase (*MTHFR*) C677T (rs1801133) gene polymorphisms are related to a growing risk of Alzheimer's disease; however, whether this association applies to mild cognitive impairment (MCI) remains unclear.

**Objective:**

We conducted this meta-analysis to evaluate the contribution of *MTHFR* C677T (rs1801133) gene variants to the risk of MCI.

**Methods:**

PubMed, Embase, Web of Science, and China National Knowledge Infrastructure databases were searched from their inception to March 21, 2021, with language restricted to English or Chinese. We used fixed or random effects to examine the association between *MTHFR* C677T (rs1801133) gene variants and MCI susceptibility. Forest plots of pooled odds ratios (ORs) and 95% confidence intervals (CIs) were generated.

**Results:**

Eight articles with 2,175 participants were included in the present meta-analysis. There was no significant association between *MTHFR* C677T (rs1801133) gene variants and MCI susceptibility under the allelic (OR, 1.318; 95% CI, 0.964–1.801; *p* = 0.084), dominant (OR, 1.296; 95% CI, 0.925–1.817; *p* = 0.132), recessive (OR, 1.397; 95% CI, 0.845–2.312; *p* = 0.193), heterozygous (OR, 1.031; 95% CI, 0.855–1.243; *p* = 0.749), or homozygous (OR, 1.506; 95% CI, 0.850–2.667; *p* = 0.160) models.

**Conclusion:**

The results suggest that *MTHFR* C677T (rs1801133) gene polymorphisms are not associated with MCI susceptibility. However, large-scale studies covering various factors are required.

## 1. Introduction

Mild cognitive impairment (MCI) is defined as a transitional stage between normal aging and dementia [1], that is, the symptomatic predementia stage, which does not fulfill the criterion for dementia diagnosis [2]. In the United States, age-related cognitive decline affects approximately 20% of people aged 70 years and older [3]. In China, the prevalence of MCI is 20.8% [4]. MCI has been attributed to numerous etiologies, such as Alzheimer's disease (AD) and psychiatric disorders [5, 6]. More than 40% of patients with MCI could revert to normal function, and 10–15% could progress to AD [7]. Additionally, diabetes and advanced age are high-risk factors for the progression of MCI to AD, and women are more risk-prone [8, 9].

Several studies have shown that a higher serum homocysteine (Hcy) concentration increases the risk of cognitive function impairment, such as AD, vascular dementia, and Parkinson's disease [10–15]. Serum Hcy levels and folate levels were related to cognitive performance, even in elderly subjects without dementia [16]. After the examination of seven polymorphisms of genes involved in Hcy metabolism, it was reported that only methylenetetrahydrofolate reductase (*MTHFR*) C677T (rs1801133) gene polymorphisms were associated with Hcy concentration [17].

MTHFR, the key enzyme of folate and Hcy metabolism [18], catalyzes the reduction of methylenetetrahydrofolate to methyltetrahydrofolate [18], which is involved in the methylation of Hcy to generate methionine to maintain the serum Hcy concentration [19]. Variants in the *MTHFR* gene, where the cytosine is replaced by thymidine (C→T) at nucleotide position 677 [20], are associated with elevated Hcy concentrations [21]. Severe MTHFR deficiency, a rare inherited disorder, can result in severe cognitive impairment [22]. Recently, a study found that omega-3 polyunsaturated fatty acids could predict cognitive impairment in those carrying the T variant after being stratified by *MTHFR* C677T (rs1801133) polymorphic allele carriage [23]. The TT genotype is associated with higher Hcy concentrations compared with the CC or CT genotypes, which means that individuals with the TT genotype who are exposed to higher serum Hcy concentrations for life should have a higher cognitive impairment than CC and CT individuals [23, 24]. Participants with the TT genotype have been reported with 46% greater odds of cognitive impairment than those with the wild CC genotype [25, 26].

Extensive studies have reported that there was an association between *MTHFR* C677T (rs1801133) gene mutations and AD susceptibility [12, 27–29]. Several studies have reported relationships between *MTHFR* C677T (rs1801133) gene polymorphisms and the risk of MCI [30–32], the symptomatic predementia stage of AD; however, the results are inconsistent. Considering these previous contradictory results, this meta-analysis was conducted to evaluate the contribution of *MTHFR* C677T (rs1801133) gene polymorphisms to MCI susceptibility with greater precision.

## 2. Materials and Methods

### 2.1. Search Strategy

The systematic review and meta-analysis adhered to the Preferred Reporting Items for Systematic Reviews and Meta-Analyses (PRISMA) guidelines [33]. We performed a meta-analysis to determine the association between *MTHFR* C677T (rs1801133) polymorphisms and MCI susceptibility. PubMed, Embase, Web of Science, and China National Knowledge Infrastructure databases were searched from their inception to March 21, 2021, by two reviewers independently. The following search term combinations were used: (MTHFR or C677T or homocysteine) and (polymorphism or variant or mutation or SNP) and (cognitive or cognition), which were adjusted based on the characteristics of the database (Supplementary Table 1). A literature search was performed without restriction to region and publication types, and publication languages were restricted to either English or Chinese.

### 2.2. Inclusion and Exclusion Criteria

Studies included in the meta-analysis met the following criteria: (1) patients with MCI, (2) *MTHFR* C677T (rs1801133) gene polymorphism as the exposure factor, (3) control group individuals with normal cognitive function, and (4) case-control study design or cohort study design. The exclusion criteria were as follows: (1) repeated publication, (2) full text unavailable, and (3) genotype distributions unavailable for both cases and controls to calculate odds ratio (ORs) or 95% confidence intervals (CIs).

### 2.3. Data Extraction and Quality Evaluation

The data extracted from the candidate studies included the first author, publication year, country, study type, ethnicity, age, sex, *MTHFR* polymorphisms, allele and genotype distribution, and sample size. “C” is used to indicate the wild-type allele while “T” indicates a mutant allele of single-nucleotide polymorphisms (SNP), respectively (C>T). We assessed the quality of eight retrospective studies, according to the Newcastle-Ottawa Scale (NOS), by examining three factors: patient selection, comparability of the study groups, and assessment of outcome [34]. A score of 0–9 (allocated as stars) was allocated to each study. Studies achieving seven or more stars were considered high quality [35], while studies with six stars or less were considered of moderate or low quality. Two reviewers conducted data extraction and literature quality evaluation independently. Any disagreements were resolved through discussion with a third investigator.

### 2.4. Statistical Analyses

All analyses were performed using Stata version 15.0 (Stata Corporation, College Station, TX, USA). Five separate analyses, the allelic model (T vs. C), dominant model (CT+TT vs. CC), recessive model (TT vs. CC+CT), heterozygous model (CT vs. CC), and homozygous model (TT vs. CC), were conducted in this meta-analysis. Pooled ORs and 95%, 95% CIs were used to assess the association between C677T (rs1801133) gene mutations and MCI susceptibility. Chi-square and *I*^2^ tests were used to examine the heterogeneity among the studies. A fixed effects model was adopted if heterogeneity was acceptable (*p* > 0.10, *I*^2^ < 50%); otherwise, a random effects model was adopted. The pooled OR was assessed using the *Z* test and defined *p* value < 0.05 as statistical significance. The chi-square test was used to determine the Hardy–Weinberg equilibrium (HWE) for the genotype frequencies, and *p* < 0.05 was considered a significant imbalance. Sensitivity analysis for this meta-analysis was conducted by sequentially omitting one study at a time to evaluate the stability of the results. We performed subgroup analysis by stratification by ethnicity (Asian and Caucasian). To evaluate any potential publication bias, the funnel plot and Egger's linear regression tests were adopted, and publication bias was defined as a *p* value of <0.05.

## 3. Results

### 3.1. Study Selection

**Figure 1:**
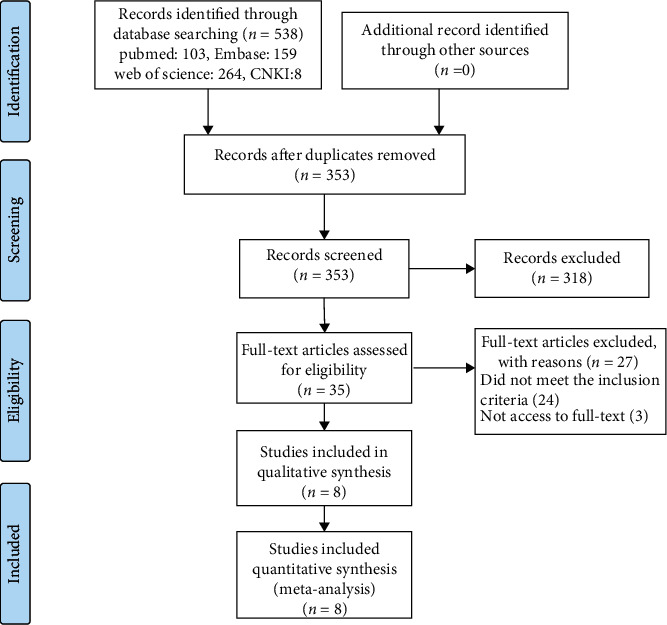
PRISMA flow diagram of the literature retrieval and selection process. CNKI: China National Knowledge Infrastructure.

A total of 538 potentially relevant studies were retrieved from the four databases. After 185 duplicates and 318 studies according to titles and abstracts were eliminated, the full texts of 35 articles were examined in detail; only eight papers [36–43] met all inclusion criteria, including four English [36–39] and four Chinese [40–43] articles. The detailed search process is illustrated in Figure 1.

### 3.2. Study Characteristics and Quality Evaluation

Eight studies were included in the present meta-analysis, involving 1,183 patients with MCI and 992 controls.  Table 1 presents the study characteristics. Of these, six were case-control studies, and two were cohort studies. Among them, participants in four studies were Asian, and those in the remaining four studies were Caucasian. The mean age of all participants ranged from 51.75 to 76.15 years. Of the eight nonrandomized studies, three studies were classified as high-quality, while five studies were classified as moderate quality. The evidence included in this meta-analysis was considered of moderate quality owing to an average NOS score of 6.25. The distributions of *MTHFR* C677T (rs1801133) genotypes and allele frequencies in MCI cases and controls are shown in Table 2. The results of the HWE test for the distribution of genotypes in the control population are also displayed in Table 2; these were not in HWE for three studies.

### 3.3. Meta-Analysis of the Association between MTHFR C677T (rs1801133) Polymorphisms and MCI Susceptibility

**Figure 2:**
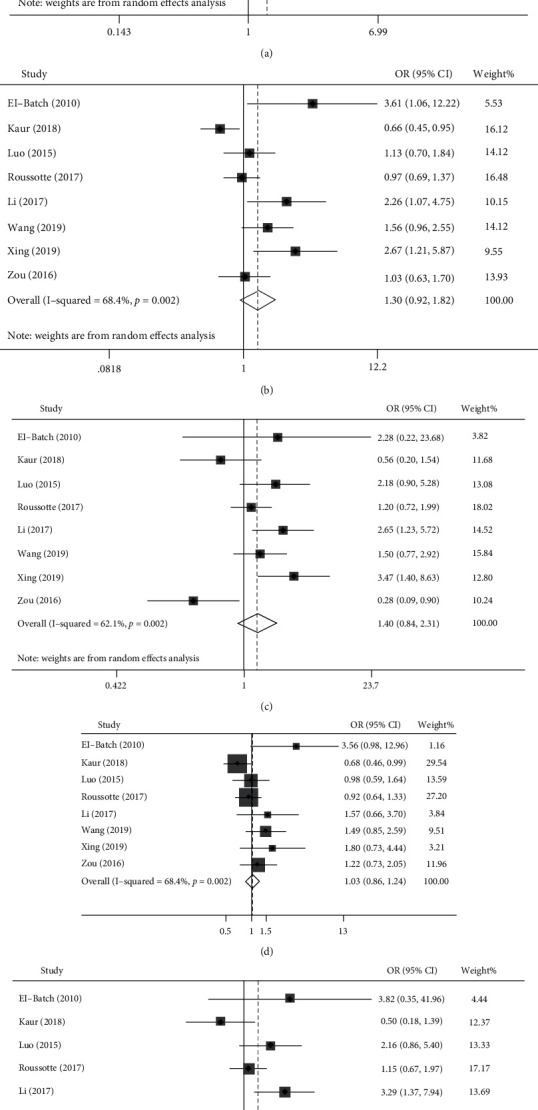
Forest plots of five gene models for the association between *MTHFR* C677T polymorphisms and mild cognitive impairment. (a) Allelic model (T vs. C), (b) dominant model (CT+TT vs.CC), (c) recessive model (TT vs. CC+CT), (d) heterozygous model (CT vs. CC), and (e) homozygous model (TT vs. CC).

The *I*^2^ was less than 50% in the heterozygous model; therefore, a fixed effects model was adopted. Random effects models were adopted in other gene models because of significant heterogeneity (*I*^2^ > 50%). Forest plots of pooled ORs with the corresponding 95% CIs are shown in Figure 2. The pooled results suggested that the *MTHFR* C677T (rs1801133) polymorphism was not significantly associated with the risk of MCI in any of the genetic frameworks, i.e., the allelic model (OR, 1.318; 95% CI, 0.964–1.801; *p* = 0.084), dominant model (OR, 1.296; 95% CI, 0.925–1.817; *p* = 0.132), recessive model (OR, 1.397; 95% CI, 0.845–2.312; *p* = 0.193), heterozygous model (OR, 1.031; 95% CI, 0.855–1.243; *p* = 0.749), or homozygous model (OR, 1.506; 95% CI, 0.850–2.667; *p* = 0.160) (Table 3).

### 3.4. Subgroup Analysis

A stratified subgroup analysis based on ethnicity was performed to investigate the exact consequences of the relationship between *MTHFR* C677T (rs1801133) gene polymorphisms and MCI susceptibility. Similar to the above results, no statistically significant association was observed in Caucasians or Asians under any genetic model between MCI risk and *MTHFR* C677T (rs1801133) genotype (Table 4).

### 3.5. Sensitivity Analysis

Sensitivity analysis was performed by omitting one study at a time to assess the robustness of the analysis to the results of individual studies. When single studies were removed one by one and the remaining studies were analyzed sequentially by meta-analysis, there was no significant change in the pooled ORs, indicating that the results were stabilized (Supplementary Figure 1).

### 3.6. Publication Bias

**Figure 3:**
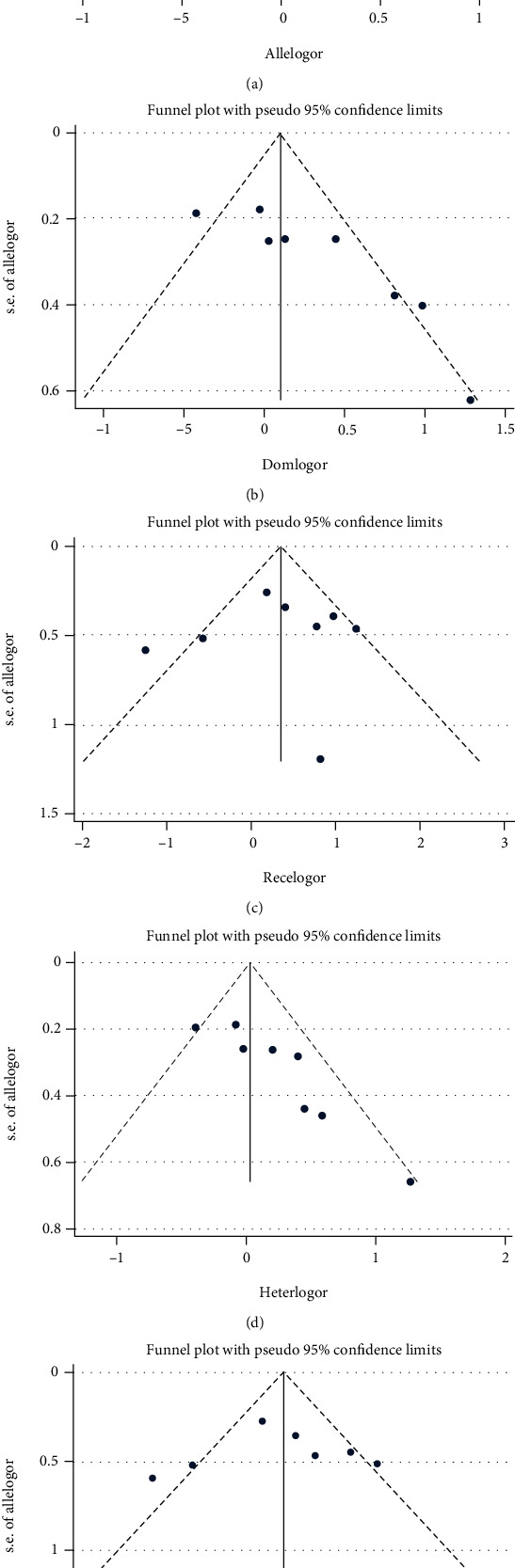
Funnel plot of five gene models for the association between *MTHFR* C677T polymorphisms and mild cognitive impairment. (a) Allelic model (T vs. C), (b) dominant model (CT+TT vs. CC), (c) recessive model (TT vs. CC+CT), (d) heterozygous model (CT vs. CC), and (e) homozygous model (TT vs. CC).

The shapes of the funnel plots were roughly symmetrical in the allelic, recessive, and homozygous models (Figure 3). Egger's regression test indicated evidence of publication bias in the dominant (*p* = 0.006) and heterozygous (*p* = 0.005) models, but no evidence of publication bias was found in the other gene models (Table 3).

## 4. Discussion

To our knowledge, this study represents the first meta-analysis of *MTHFR* C677T (rs1801133) gene polymorphisms and MCI susceptibility based on a broad range of studies involving 2,175 participants. In the current study, we did not find any statistically significant evidence that *MTHFR* C677T (rs1801133) gene variants can contribute to MCI susceptibility. No association between *MTHFR* C677T (rs1801133) gene polymorphisms and the risk of MCI was observed in the stratified analysis. The heterogeneity of the study was significant.

MTHFR, which depends on folate and vitamin B12, is a pivotal enzyme in one-carbon metabolism [44]. It has been reported that *MTHFR* C677T (rs1801133) gene variants result in lowered catalytic activity and are associated with elevated blood Hcy concentration [18, 45], which leads to a decline in cognitive function [30, 46–48]. It was found that adult cognition was associated with *MTHFR* gene polymorphisms and serum Hcy levels [49]. However, several subsequent studies have shown that *MTHFR* C677T (rs1801133) polymorphisms are not associated with individual changes in cognitive function [23, 50, 51].

Previous reports have indicated that the *MTHFR* C677T (rs1801133) gene variant could contribute to AD susceptibility [8, 27]. It has been reported that the one-carbon cycle-derived methyl donor S-adenosylmethionine influenced the key gene expression, thereby affecting cognitive function [52, 53]. First, the elevated blood Hcy concentration caused by MTHFR deficiency reduces the expression and methylation levels of Ser/Thr protein phosphatase 2A and leucine carboxylmethyltransferase 1, resulting in tau dephosphorylation, which leads to the development of AD [46, 54]. Second, it was reported that a strong correlation exists between serum Hcy and plasma amyloid beta 40 (A*β*40) concentrations, which might result in AD [55]. Additionally, the TT genotype promotes an increase in plasma Hcy, which might favor intima media thickening in patients with cognitive impairment and cause cognitive function decline [56]. Finally, it was demonstrated that brain volume deficits were up to 5–12% in the *MTHFR* T allele group with MCI [57]. MCI was considered the symptomatic predementia stage; thus, these findings could explain the relationship between *MTHFR* C677T (rs1801133) gene polymorphisms and MCI.

SNPs are DNA sequence polymorphisms resulted by single-nucleotide mutations that occur at genomic levels, which might affect the expression or activity of the encoded protein and affect its function [58]. Genetic variants were fixed at conception and tended to be specific in their associations, which means that they did not change because of environmental factors [59–61]. It must be noted that the *MTHFR* C677T (rs1801133) gene polymorphisms of the three studies were not in HWE in control groups, which might have affected our findings. Therefore, the possible association is needed to verify by a representative sample.

In interpreting the results of the current research, a few limitations should be recognized. First, the results were highly heterogeneous, which may have distorted the meta-analysis. We considered the following possible sources of this heterogeneity: (i) different study types were pooled in our research, and (ii) inconsistent results might be limited by the ethnicity of the included population. We conducted a subgroup analysis based on ethnicity, but this did not affect the final result. Second, the sample size of our research was relatively small to investigate the association between *MTHFR* C677T (rs1801133) polymorphisms and MCI risk. Third, the literature search was restricted to articles published in Chinese and English, which might introduce publication and language bias. A limited number of electronic databases were investigated, and relevant studies might have been overlooked.

In conclusion, *MTHFR* C677T (rs1801133) gene polymorphisms were not associated with MCI susceptibility based on current studies. The TT genotype did not confer an increased risk of MCI compared to the CC and CT genotypes. However, considering the small sample size and limitations of the included research, further large-scale prospective studies and randomized controlled trials are needed to confirm our findings. In particular, future studies should take into account gene–gene and gene–environment interactions, as well as other confounding factors. We hope that our results will provide background data for future MCI research and will contribute to genetic marker screening.

## Figures and Tables

**Table 1:** Study characteristics.

Author	Year	Country	Study type	Ethnicity	Age (y), mean ± sd		Gender		Sample size		NOS
					MCI	Control	MCI	Control	MCI	Control	
							Female (%)	Female (%)			
Roussotte [36]	2017	Turkey	Cohort study	Caucasian	75.14 ± 7.22	76.15 ± 4.98	128 (35.65)	94 (45.63)	359	206	7
Luo [37]	2015	China	Case-control study	Caucasian	64.32 ± 6.42	64.41 ± 6.46	55 (42.64)	55 (42.31)	129	131	7
Kaur [38]	2018	India	Cohort study	Asian	52.66 ± 10.23	51.75 ± 10.48	192 (69.57)	81 (27.18)	263	276	5
El-Batch [39]	2010	Turkey	Case-control study	Caucasian	62.86 ± 6.97	60.25 ± 4.98	13 (46.43)	15 (75.00)	28	20	7
Zou [40]	2016	China	Case-control study	Caucasian	65.76 ± 7.6	64.44 ± 6.2	55 (44.35)	54 (43.55)	124	124	6
Xing [41]	2019	China	Case-control study	Asian	65.46 ± 5.89	65.37 ± 6.98	23 (46.00)	24 (40.00)	50	60	6
Wang [42]	2019	China	Case-control study	Asian	56.6 ± 6.1	55.8 ± 6.2	73 (44.24)	58 (51.79)	165	112	6
Li [43]	2017	China	Case-control study	Asian	73.51 ± 5.17	69.02 ± 5.4	36 (55.38)	33 (52.38)	65	63	6

MCI: mild cognitive impairment; NOS: Newcastle-Ottawa scale.

**Table 2:** Genotype frequency of *MTHFR* C677T gene polymorphisms in MCI patients and the control group.

Author (year)	Genotype										HWE
	MCI patients					Control					
	C	T	CC	CT	TT	C	T	CC	CT	TT	
Roussotte (2017) [36]	455	263	149	157	53	264	148	84	96	26	*p* > 0.05
Luo (2015) [37]	175	83	62	51	16	190	72	67	56	8	*p* > 0.05
Kaur (2018) [38]	451	75	194	63	6	444	108	179	86	11	*p* > 0.05
El-Batch (2010) [39]	36	20	11	14	3	33	7	14	5	1	*p* > 0.05
Zou (2016) [40]	181	67	61	59	4	173	75	62	49	13	*p* > 0.05
Xing (2019) [41]	46	54	15	16	19	83	37	32	19	9	*p* < 0.05
Wang (2019) [42]	216	114	82	52	31	165	59	68	29	15	*p* < 0.05
Li (2017) [43]	54	76	17	20	28	77	49	28	21	14	*p* < 0.05

MCI: mild cognitive impairment; HWE: Hardy–Weinberg Equilibrium; OR: odds ratio.

**Table 3:** Meta-analysis of the association between *MTHFR* C677T polymorphisms and MCI susceptibility and Egger's test.

Comparison	*N*	Model	Pooled estimate value				Heterogeneity		*p* for Egger's test
			OR	95% CI	*Z*	*p*	*I* ^2^	*p*	
T vs. C	8	Random	1.318	0.964-1.801	1.73	0.084	78.6%	≤0.001	0.066
CT+TT vs. CC	8	Random	1.296	0.925-1.817	1.51	0.132	68.4%	0.002	0.006
TT vs. CC+CT	8	Random	1.397	0.845-2.312	1.30	0.193	62.1%	0.010	0.859
CT vs. CC	8	Fixed	1.031	0.855-1.243	0.32	0.749	46.3%	0.071	0.005
TT vs. CC	8	Random	1.506	0.850-2.667	1.40	0.160	67.1%	0.003	0.859

*N*: number of cases; OR: odds ratio; 95% CI: 95% confidence intervals; *Z*: Z test; *p*: *p* value; *I*^2^: *I*^2^ test.

**Table 4:** Subgroup analysis by ethnicity associated with *MTHFR* C677T gene polymorphisms and MCI susceptibility.

Ethnicity		Asian	Caucasian
*N*	T vs. C	4	4
OR		1.52	1.10
95% CI		0.81-2.84	0.84-1.44
*N*	CT+TT vs. CC	4	4
OR		1.49	1.11
95% CI		0.75-2.97	0.82-1.50
*N*	TT vs. CC+CT	4	4
OR		1.72	1.08
95% CI		0.86-3.43	0.47-2.46
*N*	CT vs. CC	4	4
OR		1.00	1.06
95% CI		0.75-1.31	0.82-1.37
*N*	TT vs. CC	4	4
OR		1.91	1.14
95% CI		0.82-4.46	0.50-2.59

*N*: number of cases; OR: odds ratio; 95% CI: 95% confidence interval; s.e.: standard error.

## Data Availability

PubMed, Embase, Web of Science, and China National Knowledge Infrastructure databases were searched from their inception to March 21, 2021.

## Supplementary Materials

Supplementary Materials Supplementary table 1 and figure 1 were appended in supplemental files. Supplementary table 1: search strategy. Supplementary figure 1: sensitivity analysis of five gene models for the association between MTHFR C677T polymorphisms and mild cognitive impairment. (a) Allelic model (T vs. C), (b) dominant model (CT+TT vs. CC), (c) recessive model (TT vs. CC+CT), (d) heterozygous model (CT vs. CC), and (e) homozygous model (TT vs. CC).
