# Supplementary material for: Association of Methylenetetrahydrofolate Reductase C677T Gene Polymorphisms with Mild Cognitive Impairment Susceptibility: A Systematic Review and Meta-Analysis
Source: Behav Neurol. 2021 Sep 18;2021:2962792. doi: 10.1155/2021/2962792 (PMC8464412; doi:10.1155/2021/2962792)
Supplement: Supplementary Materials — Supplementary table 1 and figure 1 were appended in supplemental files. Supplementary table 1: search strategy. Supplementary figure 1: sensitivity analysis of five gene models for the association between MTHFR C677T polymorphisms and mild cognitive impairment. (a) Allelic model (T vs. C), (b) dominant model (CT+TT vs. CC), (c) recessive model (TT vs. CC+CT), (d) heterozygous model (CT vs. CC), and (e) homozygous model (TT vs. CC). [file 2962792.f1.zip › Supplementary table 1 (1).pdf]

Supplementary Materials

Supplementary Table 1

Database	Search strategy	Total
Pubmed	((((MTHFR[Title/Abstract]) OR (C677T[Title/Abstract])) OR (Homocysteine[Title/Abstract])) AND ((((polymorphism[Title/Abstract]) OR (variant[Title/Abstract])) OR (mutation[Title/Abstract])) OR (SNP[Title/Abstract]))) AND ((cognitive[Title/Abstract]) OR (cognition[Title/Abstract]))	103
Embase	(mthfr:ab,ti OR c677t:ab,ti OR homocysteine:ab,ti) AND (polymorphism:ab,ti OR variant:ab,ti OR mutation:ab,ti OR snp:ab,ti) AND (cognitive:ab,ti OR cognition:ab,ti)	159
Web of Science	TS= (MTHFR OR C677T OR Homocysteine)<br>TS= (polymorphism OR variant OR mutation OR SNP)<br>TS= (cognitive OR cognition)<br>#1 AND #2 AND #3	264
CNKI	Topic: MTHFR or C677T	8
