# Supplementary figures and images for: Association of Methylenetetrahydrofolate Reductase C677T Gene Polymorphisms with Mild Cognitive Impairment Susceptibility: A Systematic Review and Meta-Analysis
Source: Behav Neurol. 2021 Sep 18;2021:2962792. doi: 10.1155/2021/2962792 (PMC8464412; doi:10.1155/2021/2962792)

(a)

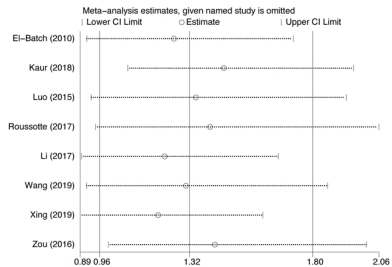

(b)

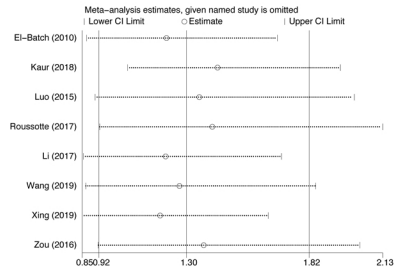

(c)

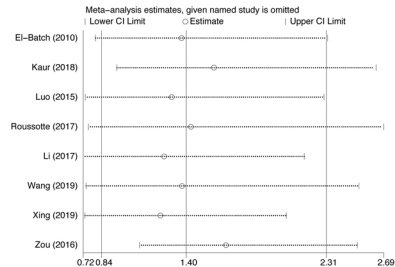

(d)

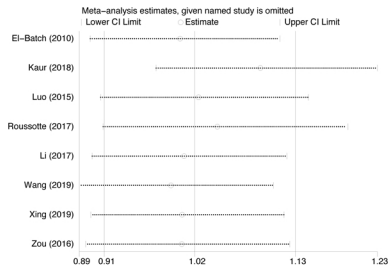

(e)

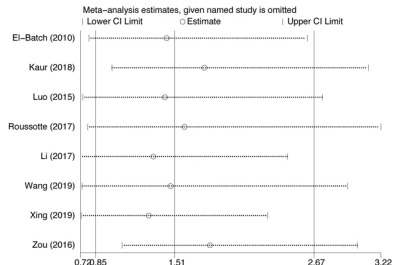

Supplement: Supplementary Materials — Supplementary table 1 and figure 1 were appended in supplemental files. Supplementary table 1: search strategy. Supplementary figure 1: sensitivity analysis of five gene models for the association between MTHFR C677T polymorphisms and mild cognitive impairment. (a) Allelic model (T vs. C), (b) dominant model (CT+TT vs. CC), (c) recessive model (TT vs. CC+CT), (d) heterozygous model (CT vs. CC), and (e) homozygous model (TT vs. CC). [file 2962792.f1.zip › Supplementary figure1 (1).pdf]
